# Whole-Transcriptome Landscape Deciphers a ceRNA-Mediated “De-Repression–Activation” Circuit Governing Continuous Estrus in Ovine

**DOI:** 10.3390/ani16182925

**Published:** 2026-09-17

**Authors:** Bo Gu, Jiayao Cui, Ruchao Tian, Limin Sun, Mingxuan Sui, Xiaowei Wei, Huaizhi Jiang

**Affiliations:** 1 College of Life Science, Jilin Normal University, Siping 136000, China; 17743110597@163.com (B.G.);; 2 Jilin Academy of Agricultural Sciences (Northeast Agricultural Research Center of China), Changchun 136100, China; 3 College of Plant Protection, Jilin Agricultural University, Changchun 130118, China; 4 College of Animal Science and Technology, Jilin Agricultural University, Changchun 130118, China

**Keywords:** year-round estrus, seasonal estrus, whole-transcriptome sequencing, ceRNA regulatory network, miRNA

## Abstract

(1) Background: Seasonal breeding constraints represent a long-standing barrier to efficient sheep production worldwide. Small Tail Han sheep, a Chinese indigenous breed that exhibits year-round estrus independent of photoperiod, offers a unique natural model for investigating the regulatory mechanisms underlying estrus cyclicity—yet the molecular basis of this remarkable trait remains largely unexplored. This study therefore aimed to compare ovarian whole-transcriptome profiles between Small Tail Han sheep (year-round estrus) and Ujumqin sheep (seasonal estrus) during estrus and diestrus. Specifically, we sought to characterize breed-specific transcriptional response strategies, identify key miRNAs associated with continuous estrus, and construct ceRNA networks that mediate ovarian estrous cycle regulation. (2) Methods: To address these objectives, we profiled ovarian transcriptomes of Small Tail Han sheep (year-round estrus) and Ujumqin sheep (seasonal estrus) during both estrus and diestrus (12 samples total) using whole-transcriptome sequencing, systematically characterizing the expression landscapes of mRNAs, miRNAs, lncRNAs and circRNAs. Through differential expression analysis, GO/KEGG functional enrichment, multi-tiered miRNA screening, and ceRNA network construction, we compared the transcriptional response strategies between the two breeds. (3) Results: Our findings revealed two unexpected and divergent patterns. Small Tail Han sheep showed 1881 differentially expressed mRNAs between estrus and diestrus, 82-fold more than the 23 in Ujumqin sheep, suggesting a broader mRNA-level transcriptional response in the year-round estrus breed. circRNAs were predominantly up-regulated (80–85%) in both breeds, while mRNAs and lncRNAs were predominantly down-regulated, uncovering a conserved “circRNA-up, mRNA-down” regulatory logic across breeds. Multi-tiered screening identified 38 “brake-releaser” miRNAs (high in diestrus, down-regulated in estrus) and 21 “accelerator” miRNAs (low in diestrus, up-regulated in estrus), which together constitute a “de-repression–activation” dual regulatory mode. ceRNA network analysis further revealed a clear functional stratification: conserved core miRNAs targeted “sensor” pathways (cAMP, PI3K-Akt, MAPK), whereas accelerator miRNAs specifically targeted “executor” pathways (estrogen signaling, progesterone-mediated oocyte maturation, Hippo signaling). Network topology analysis pinpointed miR-199a-5p, miR-204-3p and miR-199b as the core hubs bridging the two regulatory modules. (4) Conclusions: Taken together, our findings redefine the molecular basis of year-round estrus—moving beyond single-gene mutations or overexpression to an enhanced global transcriptomic responsiveness of the ovary. This work provides a fresh conceptual framework for understanding the divergence of reproductive strategies in sheep and offers actionable candidate targets for genetic improvement of continuous estrus traits.

## Simple Summary

Seasonal breeding restricts lamb production in many parts of the world, limiting the profitability and flexibility of sheep farming. Small Tail Han sheep, a Chinese breed that reproduces year-round, provide a valuable opportunity to understand how the ovary controls continuous cycling. In this study, we compared ovarian gene expression between Small Tail Han sheep and seasonally breeding Ujumqin sheep during both estrus and diestrus. Our results show that Small Tail Han ovaries change the expression of over 1800 genes between the two cycle stages, while Ujumqin ovaries change only 23—an 82-fold difference. This suggests that year-round breeders maintain highly flexible ovarian transcription, whereas seasonal breeders actively restrict it. We further identified two classes of regulatory molecules, termed “brake-releasers” and “accelerators,” that work together to control cycle transitions. Three key regulators—miR-199a-5p, miR-204-3p and miR-199b—emerged as central hubs linking these two systems. These findings shift the focus from single gene mutations to broader regulatory networks, offering new targets for breeding sheep that reproduce more consistently. This knowledge could help farmers reduce seasonal production gaps, improve lamb supply throughout the year, and enhance the resilience of sheep operations under changing environmental conditions.

## 1. Introduction

Sheep are a cornerstone of China’s livestock industry, and their reproductive efficiency directly determines farm profitability and the long-term sustainability of production systems. However, the majority of sheep breeds are seasonal breeders—their reproductive activity is tightly governed by photoperiod, with estrus and ovulation concentrated in autumn as day length progressively shortens [1,2,3]. This seasonal reproductive strategy reflects an evolutionary adaptation to high-latitude environments shaped by natural selection [4]. From a production standpoint, however, seasonal breeding restricts ewes’ reproductive activity to a narrow time window, resulting in highly synchronized lambing and precluding year-round production and continuous market supply—a constraint that critically limits the efficiency and scalability of intensive sheep operations. Elucidating the molecular mechanisms that underpin estrus cyclicity, and in particular, deciphering the differences between year-round and seasonally breeding breeds, therefore holds both fundamental scientific value and practical implications for overcoming seasonal reproductive constraints and enabling genetic improvement of reproductive efficiency in sheep.

The initiation and maintenance of the estrous cycle in sheep depend on coordinated signaling along the hypothalamic–pituitary–ovarian axis (HPOA) [5]. Photoperiodic information, transmitted from the retina to the suprachiasmatic nucleus, modulates melatonin secretion rhythms, which in turn influence the pulsatile release of gonadotropin-releasing hormone (GnRH) from hypothalamic neurons. This cascade regulates the secretion of follicle-stimulating hormone (FSH) and luteinizing hormone (LH) from the pituitary, ultimately driving follicular development, maturation, ovulation, and corpus luteum formation and regression within the ovary [6,7]. Within this intricate regulatory network, the ovary serves as the distal effector organ of the HPOA, and its intrinsic transcriptional state is critical for proper estrous cyclicity. In recent years, the rapid advancement of high-throughput sequencing technologies has enabled widespread application of transcriptomic approaches to investigate the molecular mechanisms underlying sheep reproductive traits. For instance, RNA-seq has been used to identify differentially expressed genes and lncRNAs associated with the estrous cycle in ovarian tissues of Chinese Merino and Hu sheep [8]. In Small Tail Han and Tan sheep, key genes involved in photoperiodic responses and estrogen signaling have been identified across the hypothalamus–pituitary–ovarian axis [9]. In Sunit sheep, transcriptomic analysis of the pituitary has pinpointed candidate regulatory factors for seasonal estrus [10]. While these studies have provided important clues regarding transcriptional regulation of the estrous cycle, they have predominantly focused on protein-coding genes, leaving a systematic understanding of non-coding RNAs (ncRNAs) in estrous cycle regulation largely unexplored. ncRNAs—including miRNAs, lncRNAs, and circRNAs—have recently been established as indispensable players in post-transcriptional gene regulation [11,12,13]. miRNAs typically induce mRNA degradation or translational repression through base-pairing with the 3′-UTR of target mRNAs. lncRNAs and circRNAs, in turn, can act as molecular “sponges” that competitively bind miRNAs, thereby modulating the expression of miRNA target genes and giving rise to complex ceRNA regulatory networks [14,15]. In the mammalian ovary, ncRNAs have been shown to participate in multiple key reproductive processes, including folliculogenesis, granulosa cell proliferation and apoptosis, steroidogenesis, and oocyte maturation [16,17,18]. Several recent studies have implicated specific ceRNA networks in the regulation of estrus and reproduction. In the hypothalamus of Duolang sheep, the LOC101105119-miR-106b-GNAQ ceRNA network was found to influence estrus initiation through modulation of the GnRH signaling pathway [19]. In the early-maturing Jining Grey goat breed, multiple key ceRNA networks have been identified that coordinate neuroendocrine and metabolic signals via calcium signaling and steroid biosynthesis pathways, thereby driving transitions in reproductive states [20]. In the sheep pituitary, substantial differential expression of circRNAs has been observed between estrus and anestrus, suggesting that circRNAs may participate in estrus regulation through transcriptional and post-transcriptional processes governing hormone synthesis and secretion [11]. A separate study systematically mapped the dynamic regulatory landscape across the full estrous cycle in pigs and constructed lncRNA-mRNA interaction networks, revealing widespread involvement in reproductive tissue development and hormonal responses that collectively enhance reproductive performance [21]. Despite these advances, a comprehensive understanding of how ncRNAs and their associated ceRNA networks operate at the ovarian level to regulate the estrous cycle—and whether systematic differences exist between year-round and seasonally breeding breeds—remains lacking.

Small Tail Han sheep and Ujumqin sheep are two Chinese indigenous breeds that display markedly contrasting reproductive phenotypes. Small Tail Han sheep are renowned for their year-round estrus and high fecundity, making them an ideal model for investigating the mechanisms underlying continuous reproductive activity. In contrast, Ujumqin sheep exhibit typical seasonal estrus, with reproductive activity confined largely to autumn. Both breeds belong to the Mongolian sheep lineage and share a similar genetic background, yet their reproductive strategies diverge sharply—a combination that renders them valuable genetic resources for comparative studies of estrous cycle regulation. In this study, we collected ovarian tissues from both breeds during estrus and diestrus and employed whole-transcriptome sequencing to systematically profile the expression of mRNAs, miRNAs, lncRNAs and circRNAs. Through differential expression and functional enrichment analyses, we sought to characterize the differences in ovarian transcriptional response strategies between the two breeds across the estrus-to-diestrus transition. We then applied a multi-tiered screening strategy to identify key miRNAs governing the year-round estrus trait and constructed ceRNA regulatory networks to explore how ncRNAs mediate ovarian estrous cycle regulation. Our findings are expected to offer fresh insights into the molecular control of estrous cyclicity in sheep and to provide potential molecular targets for genetic improvement of reproductive performance.

## 2. Materials and Methods

### 2.1. Sample Collection

Adult multiparous ewes used in this study were obtained from Guofeng Livestock Breeding Farm, Changling County, Jilin Province, China. A total of 12 ewes (6 Small Tail Han sheep and 6 Ujumqin sheep), all 2 years of age, raised under identical management conditions and in good health, were selected for the experiment. Prior to the formal experiment, estrous cycle synchronization was performed in three ewes from each breed by intravaginal insertion of a CIDR device (Controlled Internal Drug Release, Inter Ag Co., Ltd., Auckland, New Zealand) for 14 days, followed by intramuscular injection of pregnant mare serum gonadotropin (PMSG) (Ningbo Sansheng Biological Pharmaceutical Co., Ltd., Ningbo, China). On the following day, estrus detection was conducted using vasectomized rams, and ewes exhibiting mounting behavior were considered to be in estrus. Notably, serum samples were not collected, and hormonal assays for progesterone, estradiol, LH, and FSH were not performed in this study. Thus, the estrous cycle stage was determined based on behavioral estrus detection following CIDR + PMSG synchronization. Ovarian tissues were collected from three Small Tail Han ewes during diestrus (Sad, *n* = 3, Sad1, Sad2, Sad3) and three during estrus (So, *n* = 3, So1, So2, So3), as well as from three Ujumqin ewes during diestrus (Ud, *n* = 3, Ud1, Ud2, Ud3) and three during estrus (Uo, *n* = 3, Uo1, Uo2, Uo3) at slaughter. The excised ovaries were immediately rinsed with pre-chilled phosphate-buffered saline (PBS) to remove surface contaminants, snap-frozen in liquid nitrogen, and subsequently stored at −80 °C until further processing. All methods were carried out in accordance with relevant guidelines set by the Ministry of Agriculture of the People’s Republic of China. All experimental protocols were approved by the Ethics Committee for Science and Technology of Jilin Normal University (KJLL20260702) on 2 July 2026.

### 2.2. Total RNA Extraction, Library Construction and Sequencing

Adult Total RNA was extracted from twelve ovarian tissue samples that had been preserved in liquid nitrogen. Each sample was first pulverized using a cryogenic grinder, followed by RNA isolation with Trizol reagent (Thermo Fisher Scientific, Waltham, MA, USA). The concentration and integrity of the extracted RNA were assessed using a NanoDrop 2000 spectrophotometer (Thermo Fisher Scientific, Waltham, MA, USA) and an Agilent Bioanalyzer (Agilent Technologies, Inc., Santa Clara, CA, USA), respectively. Only samples meeting the following quality criteria were retained for subsequent analyses: RNA integrity number (RIN) ≥ 7 and absorbance ratio A260/A280 between 1.8 and 2.1. Following quality control, the qualified RNA samples were subjected to library construction and subsequent sequencing.

For lncRNA and mRNA libraries, strand-specific libraries (fr-firstrand) were constructed using the rRNA depletion method to remove ribosomal RNA. The library construction workflow is illustrated in Supplementary Figure S1. After passing quality control, the libraries were sequenced on the Illumina Novaseq™ 6000 platform (Illumina Inc., San Diego, CA, USA) with paired-end 150 bp (PE150) reads. For circRNA libraries, linear RNA digestion (RNase R^+^) was performed following rRNA depletion to further remove linear transcripts, thereby enriching circular RNAs. The construction procedure is depicted in Supplementary Figure S2. For miRNA libraries, small RNA sequencing libraries were prepared using the TruSeq Small RNA Sample Prep Kit (Illumina, San Diego, CA, USA) according to the manufacturer’s instructions. Following library preparation, the constructed libraries were sequenced on the Illumina Hiseq 2000/2500 platform (Illumina Inc., San Diego, CA, USA) with single-end 50 bp (SE50) reads.

### 2.3. Data Filtering, Alignment and Identification

To ensure accurate analytical results, raw sequencing data were preprocessed and filtered using Cutadapt (v1.10) [22] to remove adaptor sequences and low-quality reads, yielding clean data. The clean reads were then aligned to the Ovis aries reference genome (ARS-UI_Ramb_v3.0) for subsequent analyses.

For lncRNA identification, transcripts were assembled using StringTie (v2.1.6) [23,24,25]. Known mRNAs and transcripts shorter than 200 bp were discarded, and transcripts with read coverage >3 and at least one exon were retained for coding potential prediction using CPC (Coding Potential Calculator) (v0.9-r2) [26] and CNCI (Coding-Non-Coding Index) (v2.0) [27]. Transcripts predicted to have protein-coding potential by either tool were classified as novel mRNAs and excluded. The intersection of transcripts predicted as non-coding by both CPC and CNCI was designated as novel lncRNAs.

For circRNA identification, in contrast to mRNA and lncRNA analyses, the analysis focused on reads that failed to align to the reference genome in a conventional manner. From these unaligned reads, back-spliced junctions (BSJs) were identified, and circRNAs were detected and quantified based on reads spanning the BSJs, as illustrated in Supplementary Figure S3.

For miRNA identification, ACGT101-miR (v4.2) was employed following a multi-step filtering pipeline: (1) removal of 3′ adaptor sequences and low-quality reads to obtain clean data; (2) retention of small RNAs with lengths ranging from 18 to 26 nt; (3) alignment of the remaining sequences against mRNA, RFam, and Repbase databases (excluding miRNA sequences) to filter out non-miRNA sequences; and (4) alignment of the filtered reads against miRNA precursors and the reference genome for miRNA identification.

### 2.4. Differential Expression Analysis

For mRNAs and lncRNAs, expression levels in the Uo vs. Ud and So vs. Sad comparison groups were normalized using the FPKM method, and differential expression analysis between groups was performed using DESeq2. DEGs and differentially expressed lncRNAs (DELs) were identified with the threshold of |log_2_fc| ≥ 1 and *padj* < 0.05.

For circRNAs, identification and quantification relied on BSJ reads. Unlike the normalization approaches used for mRNAs and lncRNAs, circRNA expression levels were quantified using the srpbm method. Differential expression analysis between groups was conducted using DESeq2, with the threshold set at |log_2_fc| ≥ 1 and *padj* < 0.05.

For miRNAs, differential expression analysis between groups was performed using DESeq2. Normalized expression levels were calculated as TPM values, and fold changes were evaluated. The *p*-values were adjusted for false discovery rate (FDR) using the Benjamini and Hochberg method. Differentially expressed miRNAs (DEMs) were identified with the threshold of *padj* < 0.05 and |log_2_fc| > 1.

### 2.5. Target Gene Prediction and Bioinformatics Analysis

Target genes of significantly DEMs were predicted using TargetScan (v5.0) and miRanda (v3.3a). The intersection of the predictions from both software packages was retained as the final target gene set for each miRNA, with thresholds set at TargetScan_score ≥ 50 and miranda_Energy < −10. The miRNA–target gene co-expression networks were then visualized using Cytoscape (v3.10.1).

Gene Ontology (GO) and Kyoto Encyclopedia of Genes and Genomes (KEGG) enrichment analyses were performed on the predicted target genes using the DAVID database (https://davidbioinformatics.nih.gov/, accessed on 15 April 2026) to explore their potential molecular functions. For GO analysis, hypergeometric testing was applied to identify significantly enriched terms, with *p* < 0.05 set as the significance threshold. The enriched terms covered three ontological categories: biological process (BP), molecular function (MF), and cellular component (CC). For pathway analysis, KEGG pathway enrichment was similarly conducted using hypergeometric testing, with each KEGG pathway term treated as a basic unit for identifying significantly enriched signaling and metabolic pathways associated with the target genes.

### 2.6. Screening of Key Factors Regulating Year-Round Estrus Versus Seasonal Estrus

To screen for key molecules underlying the differential estrus phenotypes between Small Tail Han sheep (year-round estrus) and Ujumqin sheep (seasonal estrus), we adopted a multi-step sequential screening strategy. First, we retained miRNAs whose target genes were significantly enriched in pathways related to estrus regulation, including reproduction, ovarian function, hormone synthesis, oocyte maturation, and photoperiod signal transduction. On this basis, we further distinguished breed-specific DEMs between the two breeds. The intersection of differentially expressed molecules in Small Tail Han sheep (estrus vs. diestrus, designated S_DE) and those in Ujumqin sheep (U_DE) was defined as estrus-cycle-conserved core molecules (S_DE ∩ U_DE). Molecules present in S_DE but not in U_DE were defined as Small Tail Han-specific molecules (S_DE − U_DE), and those present in U_DE but not in S_DE were defined as Ujumqin-specific molecules (U_DE − S_DE).

To precisely identify core factors governing the year-round estrus trait, we retained only those genes that responded to the estrous cycle exclusively in Small Tail Han sheep (i.e., S_DE − U_DE) as the primary candidate gene pool. We then further screened for genes that exhibited basal expression differences between the two breeds during diestrus (Sad vs. Ud). Finally, the intersection of the above two gene sets was taken. This intersection was regarded as “genetically anchored estrus-responsive core genes,” because the genes identified here not only exhibited inherent expression differences between breeds but also underwent dynamic changes with the estrous cycle in the year-round estrus breed, thereby directly linking genetic background to cyclicity plasticity. To further refine the results, we stratified the intersecting genes according to their direction of expression change (up- or down-regulated) to distinguish two regulatory modes: “de-repression drivers” (high expression in diestrus and down-regulated in estrus) and “activation drivers” (low expression in diestrus and up-regulated in estrus).

### 2.7. Construction of ceRNA Regulatory Networks

To explore the molecular mechanisms by which estrus regulates reproductive function in sheep, we constructed ceRNA networks based on the whole-transcriptome sequencing data. The pipeline proceeded as follows. First, Pearson correlation analysis was performed using miRNA and transcriptome expression profiles to calculate miRNA–mRNA interactions, and negatively correlated DEM–DEG pairs were retained. Similarly, the circRNA and lncRNA targets of miRNAs were predicted using TargetScan (v5.0) and miRanda (v3.3a), and the intersections (thresholds: TargetScan_score ≥ 50 and miranda_Energy < −10) were used to identify miRNA–lncRNA and miRNA–circRNA interactions. For the ceRNA network, Pearson correlation coefficients (cor) were calculated, and pairs with cor < −0.4 and *p* < 0.05 were retained as negatively correlated lncRNA–miRNA, circRNA–miRNA, and miRNA–mRNA pairs. The ceRNA regulatory networks were constructed by assembling all co-expressed competitive triples identified, yielding lncRNA (circRNA)–miRNA–mRNA interaction networks, which were visualized using Cytoscape (v3.10.1).

### 2.8. RT-qPCR Validation

To validate the reliability of the RNA-Seq results, three lncRNAs, three circRNAs, and three miRNAs were randomly selected from each of the two comparison groups for reverse transcription quantitative real-time PCR (RT-qPCR) validation. Gene-specific primers were designed using Primer Premier 5.0 (PREMIER Biosoft International, Palo Alto, CA, USA; https://www.premierbiosoft.com/), with β-actin serving as the internal reference gene. Since mature miRNA sequences are short and lack poly(A) tails, they cannot be primed using the same approach as mRNA primers. Therefore, stem-loop reverse transcription primers for miRNAs were designed using the sRNAPrimerDB software (v1.0), with U6 used as the internal control. Primer sequences are provided in Supplementary Table S1. Relative expression levels of the target molecules were calculated using the 2^−ΔΔCt^ method, and statistical significance was assessed by one-way analysis of variance (ANOVA) using SPSS 18.0 software (IBM Corp., Armonk, NY, USA; https://www.ibm.com/products/spss-statistics, accessed on 20 May 2026).

## 3. Results

### 3.1. Data Output and Quality Assessment of Whole-Transcriptome Sequencing

A total of 12 ovarian tissue samples were collected from Small Tail Han sheep (So1, So2, So3, Sad1, Sad2, Sad3) and Ujumqin sheep (Uo1, Uo2, Uo3, Ud1, Ud2, Ud3). The total RNA quality assessment results are presented in Supplementary Table S2, and all samples met the requirements for subsequent experiments in terms of RNA concentration and integrity. For strand-specific libraries of long transcripts, after stringent quality control and low-quality data filtering, an average of over 70 million valid reads were obtained per sample across all groups, providing sufficient coverage depth for quantitative and structural analysis of full-length transcripts. For sRNA libraries, approximately 7 million valid reads were obtained per sample, which fully met the requirements for quantification of known miRNAs and prediction of novel miRNAs, and were consistent with standard data volumes for small RNA sequencing.

Quality inspection of the sequencing data revealed that the mean Q30 base percentage across all libraries was as high as 96%, and the GC content distribution was consistently above 45%, indicating stable sequencing performance, high base-calling accuracy, and no apparent sequence bias. In terms of reference genome alignment, the mapping efficiency of the strand-specific libraries ranged from 92.07% to 94.57%, ensuring effective coverage for long transcript analysis. Meanwhile, the alignment rates of the sRNA libraries all exceeded 98.89%, satisfying the requirements for small RNA identification. Detailed quality metrics are provided in Supplementary Tables S3 and S4.

### 3.2. Identification and Basic Structural Features of ncRNAs

In this study, 12 ovarian samples (Uo1–3, Ud1–3, So1–3, and Sad1–3) were subjected to systematic identification of ncRNAs. The numbers of known lncRNAs identified in each sample were 4381, 4697, 4259, 4061, 3986, 4127, 4041, 4627, 4362, 4324, 4348, and 4374, respectively, while the numbers of novel lncRNAs were 3875, 3999, 3762, 3771, 3622, 3774, 3688, 3981, 3824, 4126, 4088, and 3914, respectively. The distribution of lncRNA categories across groups is shown in Supplementary Figure S4. All identified lncRNA transcripts were localized to exonic regions of protein-coding genes. The majority of lncRNAs contained two exons (Supplementary Figure S5a), and transcript lengths were predominantly >1000 bp (Supplementary Figure S5b). The ORF length distribution revealed that the 100–200 bp interval accounted for the highest proportion (Supplementary Figure S5c). These basic features provided a reliable data foundation for subsequent differential expression and functional enrichment analyses.

A total of 14,015, 12,880, 11,645, 8564, 11,248, 8435, 9425, 12,323, 10,507, 6533, 7025, and 7645 circRNAs were identified in the four groups of ovarian samples (Uo1–3, Ud1–3, So1–3, and Sad1–3), respectively. After normalization, circRNA expression levels were generally comparable across groups (Supplementary Figure S6a). The length distribution of these circRNAs (Supplementary Figure S6b) was predominantly concentrated in the >1000 nt interval, which is largely consistent with the typical circRNA length ranges reported in mammals (e.g., predominantly 500–2000 nt in cattle and pigs). In terms of genomic origin, exonic circRNAs constituted the largest proportion, followed by intronic ciRNAs and intergenic circRNAs (Supplementary Figure S6c). The widespread detection of circRNAs and the differential distribution among their subtypes suggest that they may participate in the regulation of the ovarian estrous cycle through competing endogenous RNA mechanisms or by modulating host gene expression.

Bioinformatics analysis was performed on the small RNA sequencing data. Clean reads aligned to the reference genome were integrated and annotated, and unique annotation results for each sRNA sequence were extracted. As shown in Supplementary Figure S7a, the total rRNA content across all ovarian libraries approached zero, far below the 15% quality control threshold reported in the literature, indicating good sample quality. Further analysis revealed (Supplementary Figure S7b) that both known and predicted miRNAs exhibited a clear 5′-terminal uridine bias. The length distribution patterns were similar across samples (Supplementary Figure S7c), with peaks concentrated at 20–24 nt, consistent with the canonical molecular features of miRNAs.

### 3.3. Differential Expression Analysis

**Figure 1:**
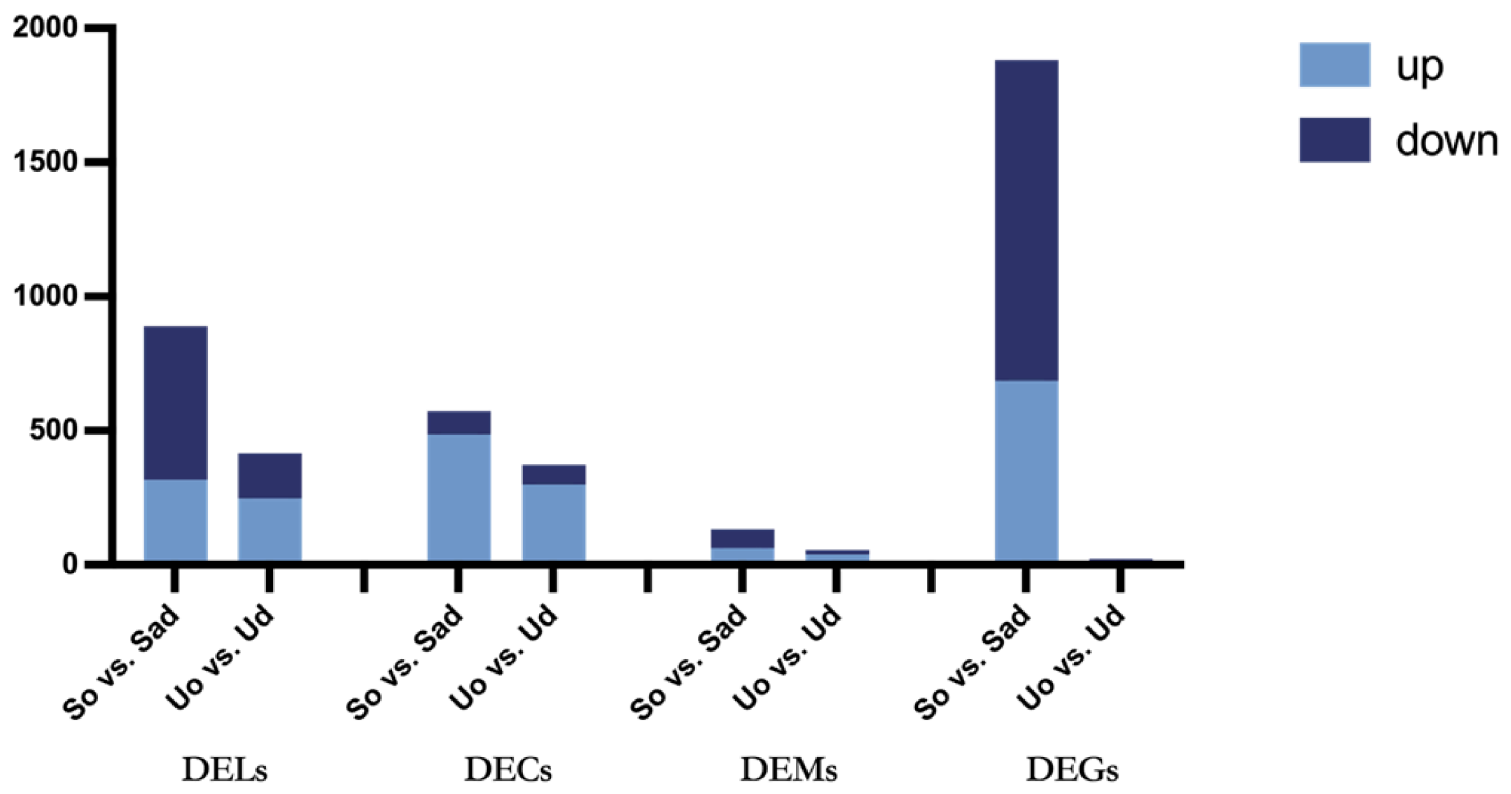
Statistics of differentially expressed transcripts in the two comparison groups. DELs: differentially expressed lncRNAs; DECs: differentially expressed circRNAs; DEMs: differentially expressed miRNAs; DEGs: differentially expressed mRNAs.

Differential expression analysis revealed that, in the So vs. Sad comparison, a total of 3475 differentially expressed transcripts were identified, comprising 573 circRNAs, 889 lncRNAs, 132 miRNAs, and 1881 mRNAs (Figure 1). Among these, 1550 transcripts were up-regulated and 1925 were down-regulated, indicating an overall predominance of down-regulation. However, the directional trends varied markedly among RNA types: circRNAs were predominantly up-regulated (485 up vs. 88 down), whereas lncRNAs and mRNAs showed the opposite pattern, with down-regulated transcripts accounting for 64.2% (571/889) and 63.6% (1196/1881) of their respective totals. miRNAs exhibited a more balanced trend, with a slight predominance of down-regulation (70 down vs. 62 up). In the Uo vs. Ud comparison, the total number of differentially expressed transcripts dropped sharply to 868, including 373 circRNAs, 416 lncRNAs, 56 miRNAs, and only 23 mRNAs. In contrast, circRNAs remained predominantly up-regulated (299 up vs. 74 down); lncRNAs also showed an up-regulation bias (248 up vs. 168 down); miRNAs shifted to an up-regulation predominance (39 up vs. 17 down); and although the number of differentially expressed mRNAs was extremely low (23), the down-regulated ones (15) slightly outnumbered the up-regulated ones (8). The differential expression results are visualized as volcano plots, as shown in Supplementary Figures S8 and S9. Collectively, these results indicated that the So vs. Sad contrast elicited far more pronounced changes in protein-coding genes than the Uo vs. Ud contrast, in which the mRNA-level response was nearly quiescent, suggesting a fundamental difference in the breadth and depth of gene expression regulation between the two comparisons. These findings provide a critical data foundation for further dissection of the molecular basis of year-round estrus in Small Tail Han sheep.

### 3.4. Bioinformatics Analysis

**Figure 2:**
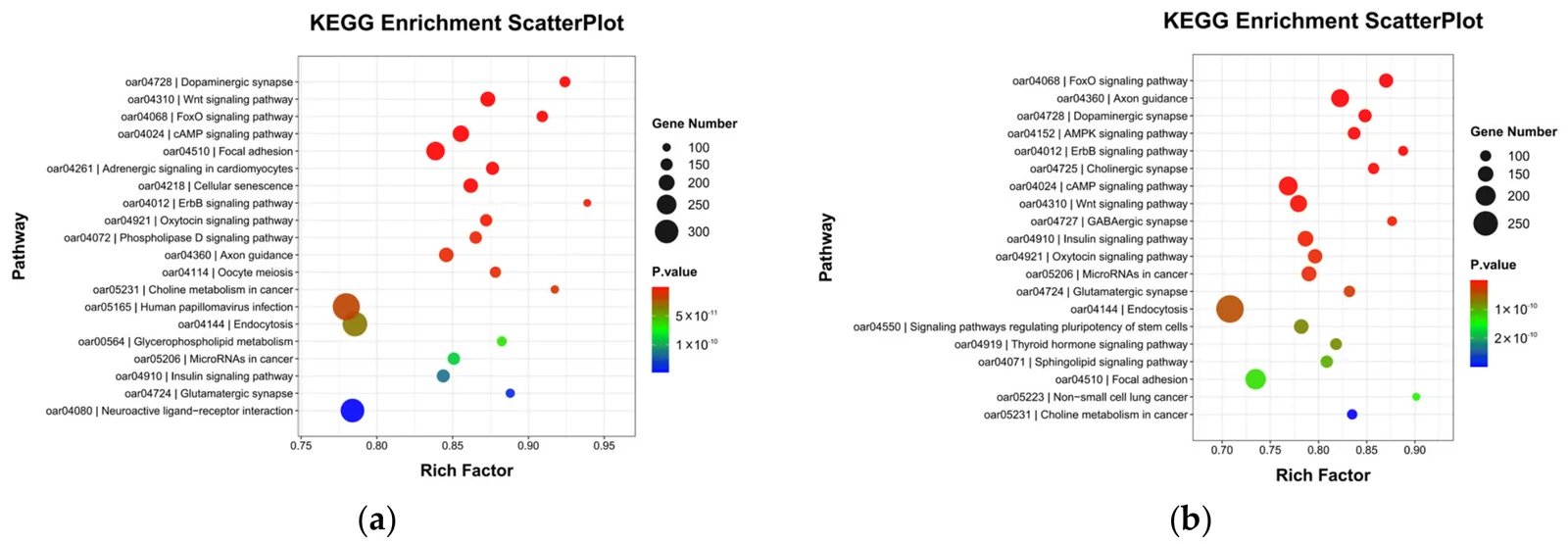
KEGG pathway enrichment analysis: (**a**) So vs. Sad; (**b**) Uo vs. Ud.

Functional enrichment analysis revealed that, in the So vs. Sad comparison, the predicted target genes of DEMs were significantly enriched in KEGG pathways including the Wnt signaling pathway, FoxO signaling pathway, cAMP signaling pathway, Oxytocin signaling pathway, and Oocyte meiosis (Figure 2a). These pathways are all well-established regulators of mammalian follicular development, steroidogenesis, and oocyte maturation, and have been repeatedly documented in transcriptomic studies of the ovine estrous cycle. Notably, the enrichment of the Oxytocin signaling pathway and Oocyte meiosis suggests that Small Tail Han sheep may employ differential miRNA expression to exert more refined post-transcriptional regulation over target genes involved in pre-ovulatory hormonal responses and meiotic resumption during estrus.

In the Uo vs. Ud comparison, the predicted target genes were significantly enriched in the FoxO signaling pathway, AMPK signaling pathway, ErbB signaling pathway, and cAMP signaling pathway (Figure 2b). Pathways commonly enriched in both comparisons included the Wnt, FoxO, and cAMP signaling pathways, indicating that miRNAs in both breeds target genes belonging to a shared set of core regulatory modules during the estrus-to-diestrus transition. However, notable differences were also observed in the pathway enrichment profiles between the two groups, suggesting that the year-round estrus breed recruits a broader spectrum of signaling pathway resources during estrus, with particularly enhanced miRNA-mediated targeting specificity toward pathways related to oocyte maturation and hormonal responsiveness.

**Figure 3:**
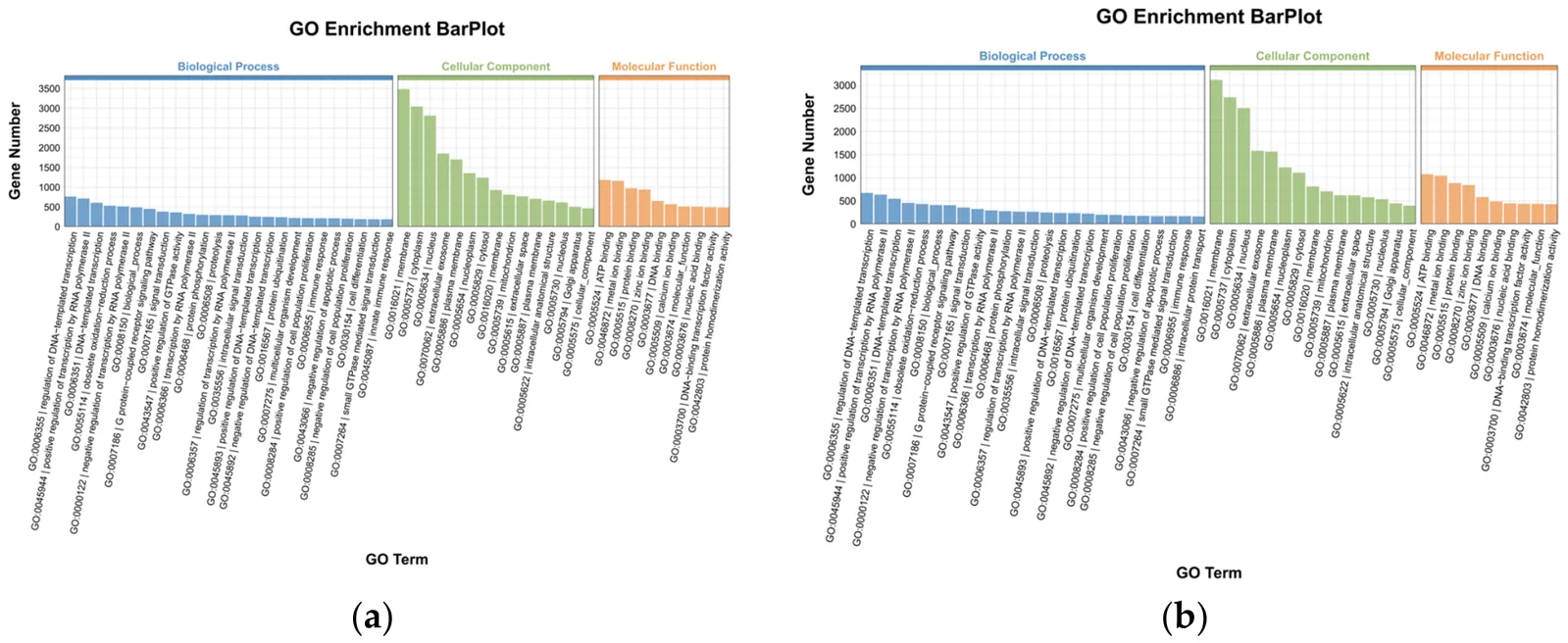
GO annotation analysis: (**a**) So vs. Sad; (**b**) Uo vs. Ud.

GO annotation analysis revealed that the significant terms at the three GO levels largely overlapped between the two comparison groups, as shown in Figure 3. At BP level, the top enriched terms for target genes in both groups were highly consistent, with transcription by RNA polymerase II and intracellular signal transduction being the core terms. At CC level, target genes in both groups were mainly localized to the nucleoplasm, cytoplasm, and similar compartments. At MF level, shared significant terms included DNA-binding transcription factor activity and metal ion binding. These common GO terms corresponded with the FoxO and cAMP signaling modules that were also shared between the two breeds in the preceding KEGG pathway analysis, jointly indicating that during the estrous-to-diestrous transition, the genes targeted by DEMs in both breeds consistently tend to be involved in fundamental transcriptional regulation, intracellular signal transduction, and ion-binding functions. In addition, differences in GO annotations between the two comparison groups were mainly reflected in a few minor terms, suggesting potential regulatory divergence between the two breeds: in the continuously estrous breed, miRNAs during estrus additionally target genes related to apoptosis and chromatin remodeling, whereas in the seasonally estrous breed, target genes are relatively more focused on functions associated with cytoplasmic protein–protein interactions.

### 3.5. Screening of Key Factors Regulating Year-Round Estrus

**Figure 4:**
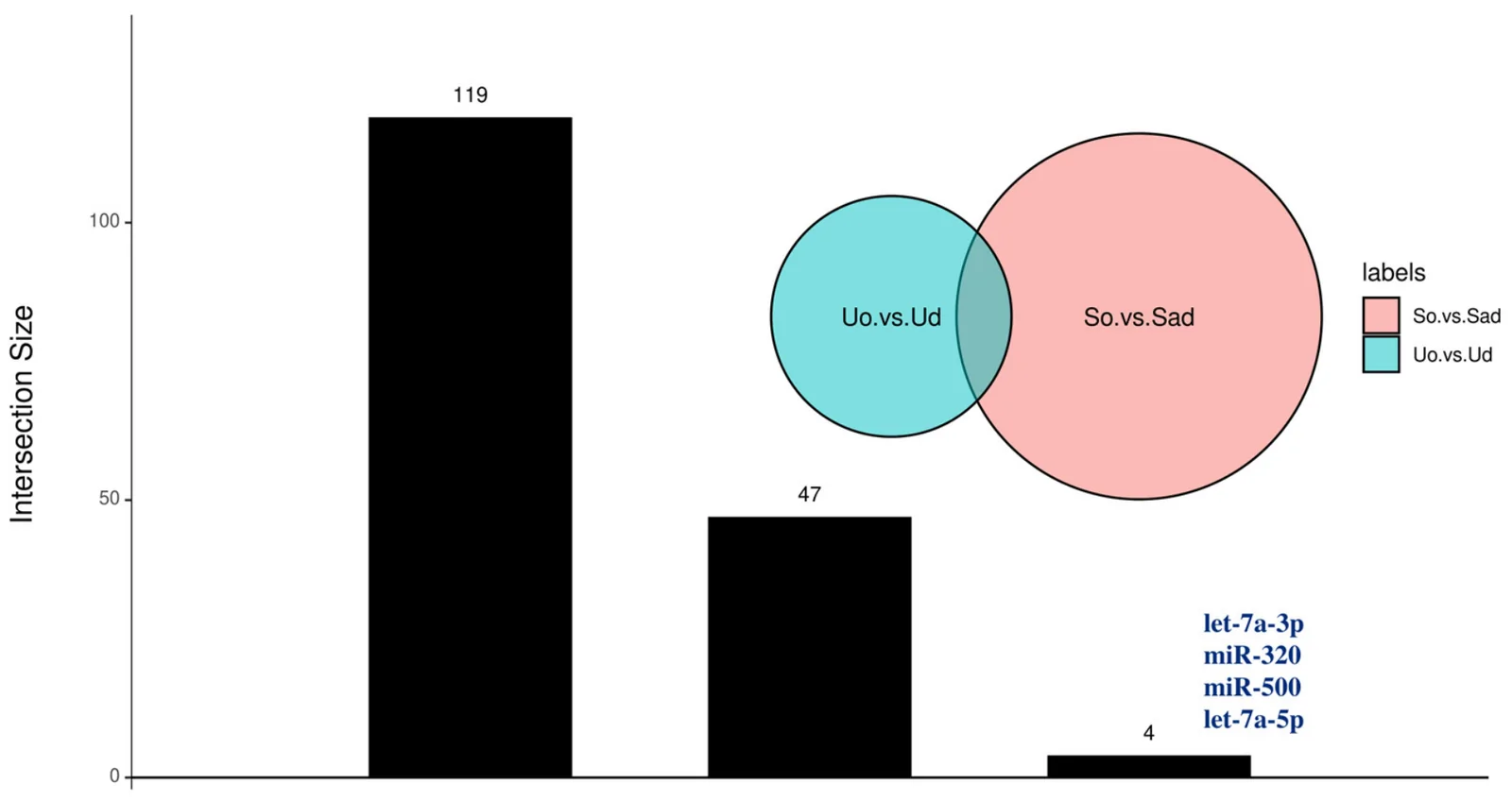
Venn diagram of DEMs between So vs. Sad and Uo vs. Ud.

To elucidate the molecular regulatory mechanism underlying permanent estrus in Small Tail Han sheep, this study employed a multi-level screening strategy to classify and analyze DEMs. The results showed that four conserved core molecules across the estrous cycle (S_DE ∩ U_DE) were identified, namely let-7a-3p, miR-320a, miR-500, and let-7a-5p, suggesting that these molecules may participate in the fundamental estrous regulatory pathways common to both breeds (Figure 4).

**Figure 5:**
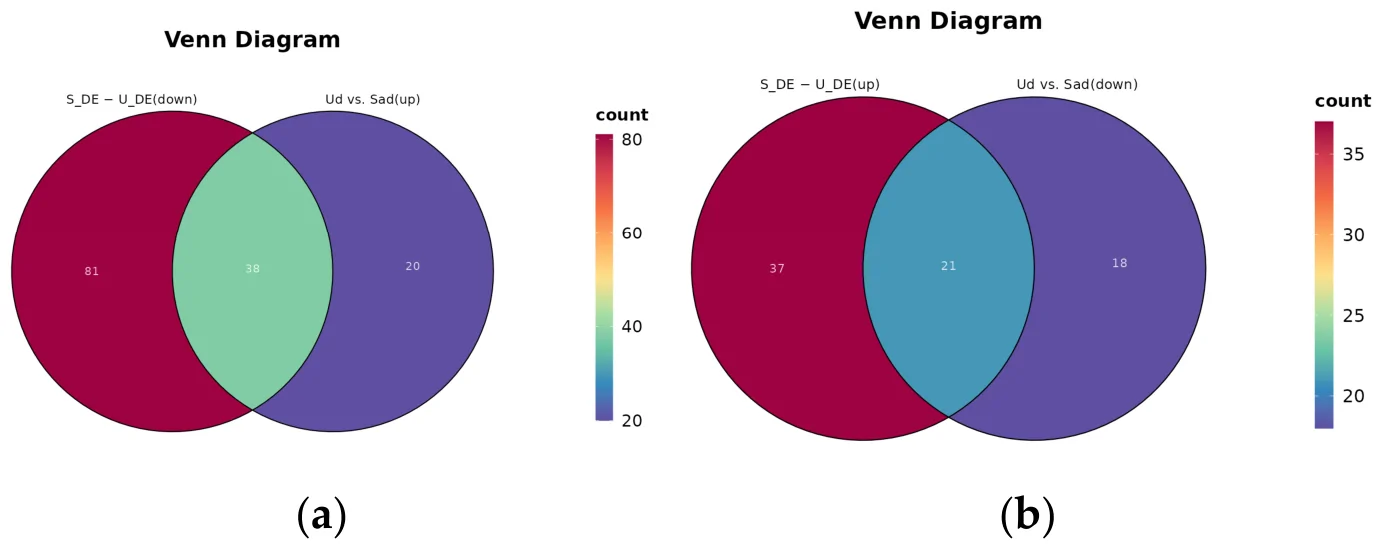
Venn Diagram of DEMs: (**a**) Brake-releasers; (**b**) accelerator-pressers.

Further, to screen for specific molecules driving the permanent estrus trait, we took the molecules that responded to the estrous cycle exclusively in the permanently estrous breed (S_DE − U_DE) as a candidate pool and intersected them with the molecules showing basal expression differences between the two breeds during diestrus (Sad vs. Ud). Based on their expression patterns, we propose a hypothetical ‘de-repression–activation’ model. The 38 ‘brake-releaser’ miRNAs (Figure 5a) were highly expressed during diestrus and down-regulated during estrus, whereas the 21 ‘accelerator’ miRNAs (Figure 5b) were low during diestrus and up-regulated during estrus. These results suggest that the maintenance of permanent estrus may rely on the synergistic action of two regulatory strategies—”de-inhibition” and “active activation”—among which the 38 brake-releasing miRNAs may constitute the core regulatory network for relieving the estrous quiescence state, while the 21 activating miRNAs may play positive driving roles in the initiation of estrus. The synergistic interplay between these two classes of molecules provides new insights into the molecular regulatory mechanisms underlying permanent estrus in Small Tail Han sheep.

### 3.6. Construction of a ceRNA Regulatory Network Based on Conserved Core miRNAs

**Figure 6:**
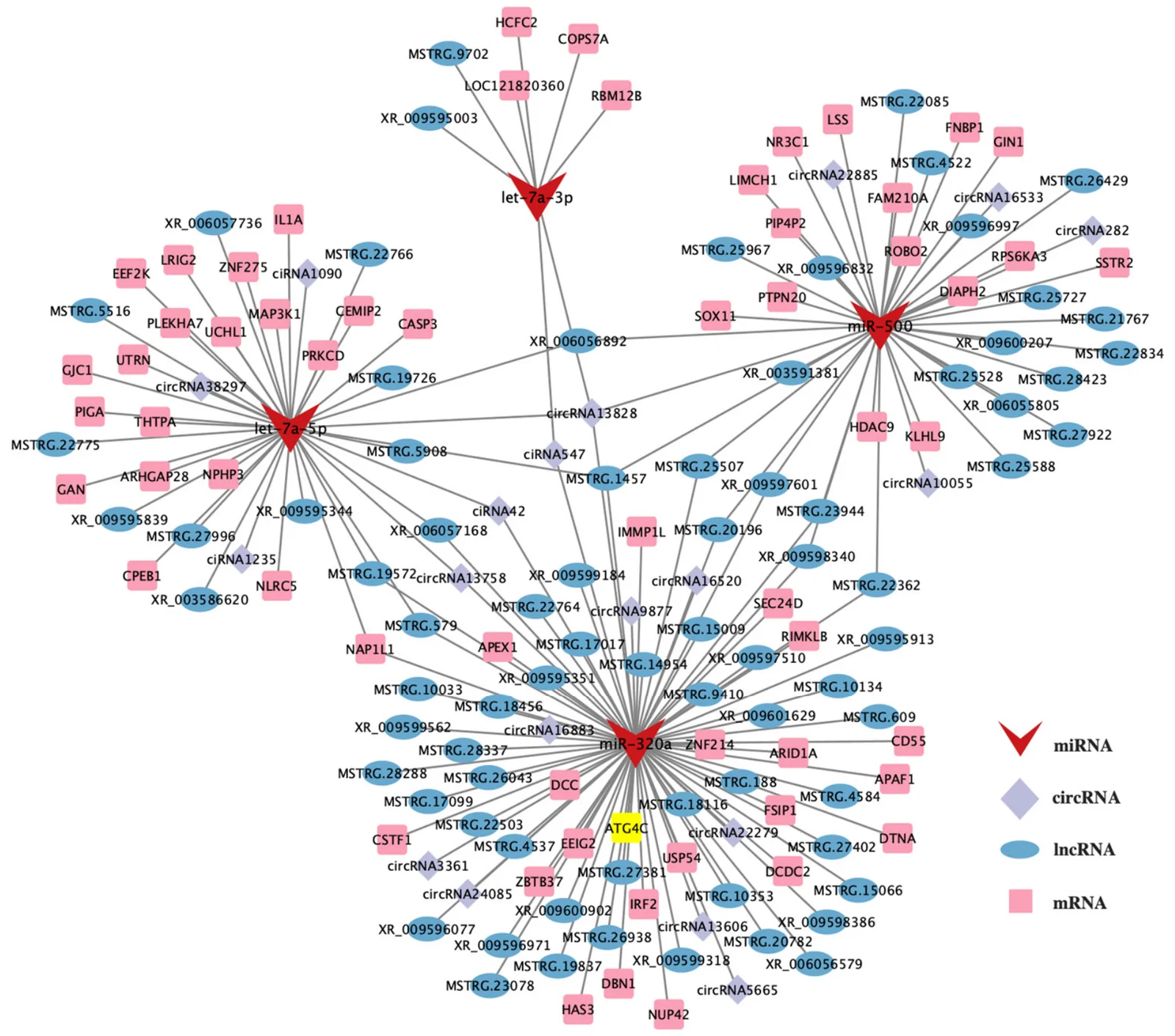
ceRNA regulatory network based on conserved core miRNAs.

To further elucidate the potential molecular mechanisms by which estrous-cycle-conserved core miRNAs participate in the regulation of permanent estrus, we systematically predicted the interacting circRNAs, lncRNAs, and target mRNAs for the four identified conserved core miRNAs (let-7a-3p, miR-320a, miR-500, and let-7a-5p) based on whole-transcriptome sequencing data, and constructed a lncRNA(circRNA)–miRNA–mRNA ceRNA regulatory network, as shown in Figure 6. The prediction results showed that the four conserved core miRNAs corresponded to a total of 25 circRNAs, 91 lncRNAs, and 60 mRNAs. Visualization analysis of the ceRNA network revealed that the same miRNA could be co-regulated by multiple circRNAs and lncRNAs, while a single circRNA or lncRNA could also simultaneously target multiple miRNAs, forming a complex multi-layered regulatory network. Notably, let-7a-3p and let-7a-5p are members of the let-7 family, and the circRNAs and lncRNAs targeted by these two miRNAs partially overlapped, suggesting that the let-7 family may exert synergistic functions in the regulation of the estrous cycle.

**Figure 7:**
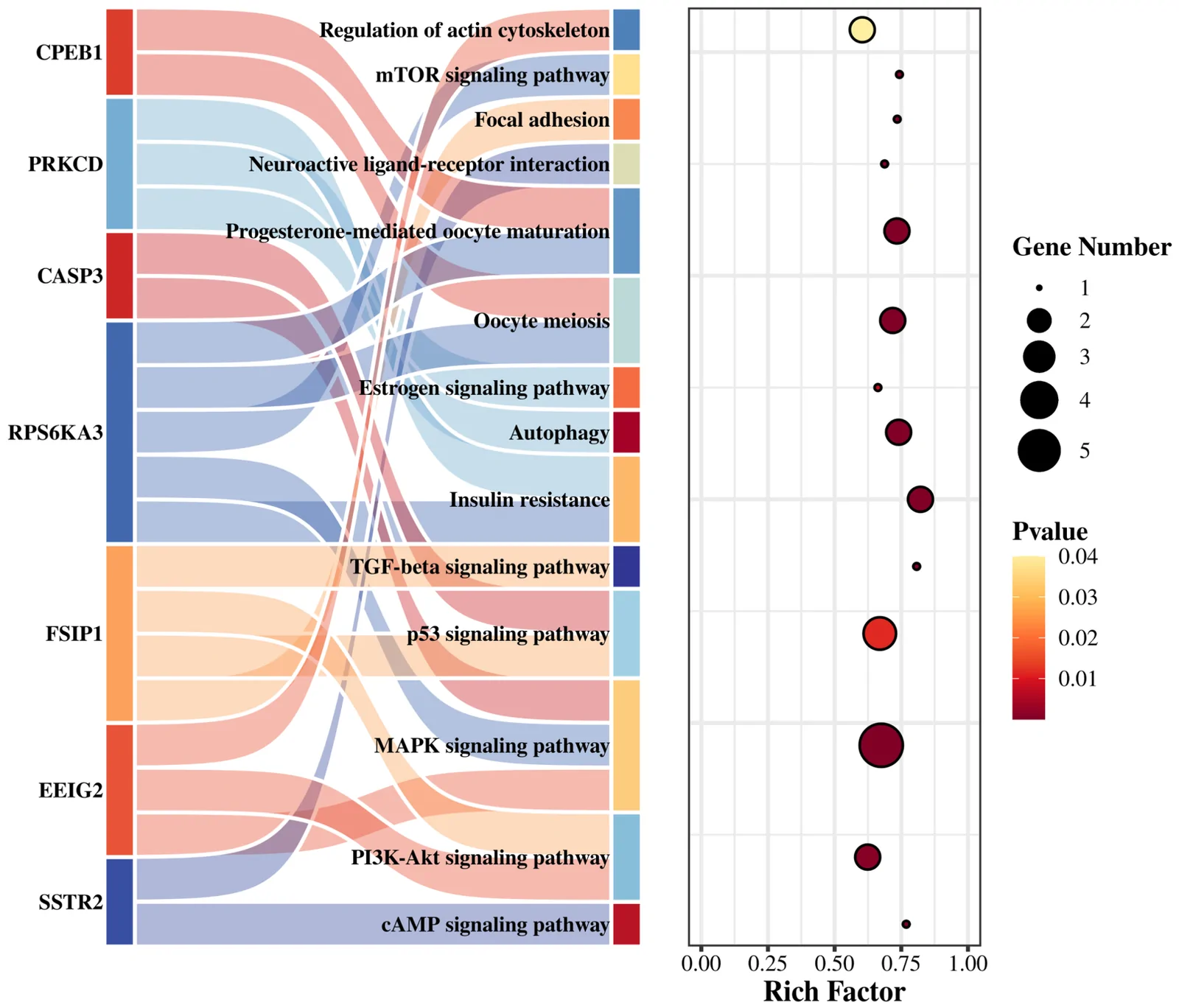
Bubble Map of Target Gene KEGG-Enriched Sankey.

Functional enrichment analysis showed that the target genes in the network were significantly enriched in multiple pathways closely associated with follicular development and ovulation regulation, including the cAMP signaling pathway, PI3K-Akt signaling pathway, MAPK signaling pathway, and Oocyte meiosis, as shown in Figure 7. Furthermore, based on connectivity degree, we screened for core regulatory nodes and identified several potential regulatory axes with high connectivity, such as circRNA13828–let-7a-5p–PRKCD, lncRNA XR_006056892–miR-500–RPS6KA3, and lncRNA MSTRG.1457–miR-320a–FSIP1. These core axes may constitute key pathways of the conserved regulatory network across the estrous cycle, providing important candidates for subsequent functional validation.

### 3.7. Construction of a ceRNA Regulatory Network Based on Accelerator-Type miRNAs

**Figure 8:**
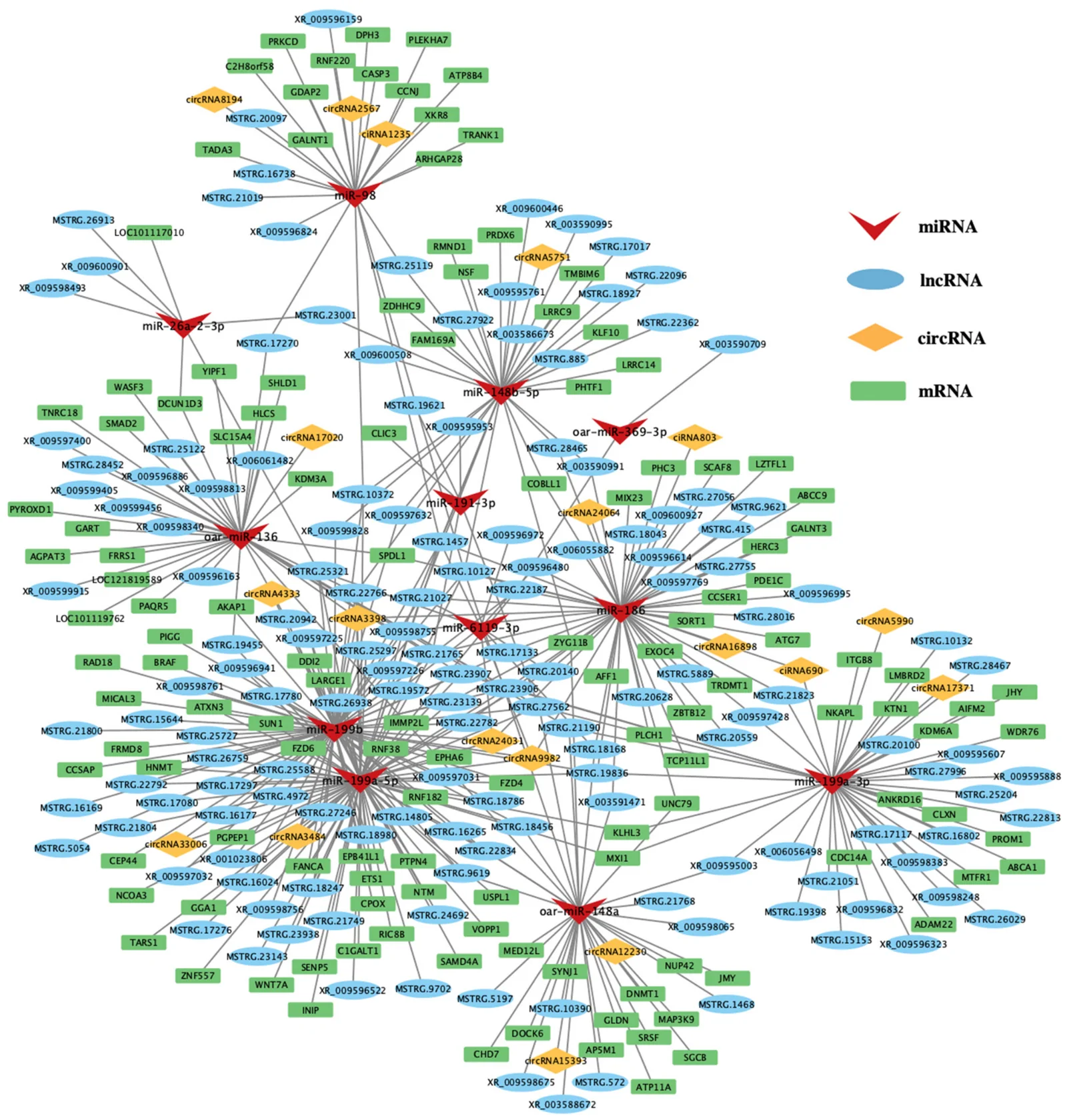
ceRNA regulatory network based on accelerator-type miRNAs.

To further elucidate the molecular mechanisms by which breed-specific accelerator-type miRNAs (low expression during diestrus and significantly upregulated during estrus) promote the initiation of estrus in the permanently estrous breed, we selected 12 representative miRNAs from the 21 previously identified activating miRNAs based on the significance of target gene enrichment and their degree of association with reproductive pathways. These included miR-26a-2-3p, oar-miR-148a, oar-miR-369-3p, oar-miR-136, miR-199a-5p, miR-191-3p, miR-148b-5p, miR-98, miR-186, miR-199b, miR-199a-3p, and miR-6119-3p, and a ceRNA network was constructed for them. This ceRNA network incorporated a total of 256 lncRNAs, 31 circRNAs, and 164 mRNAs (Figure 8). Visualization analysis of the network revealed that multiple miRNAs shared a large number of common lncRNA and circRNA sponges, forming a dense network of competitive binding interactions.

**Figure 9:**
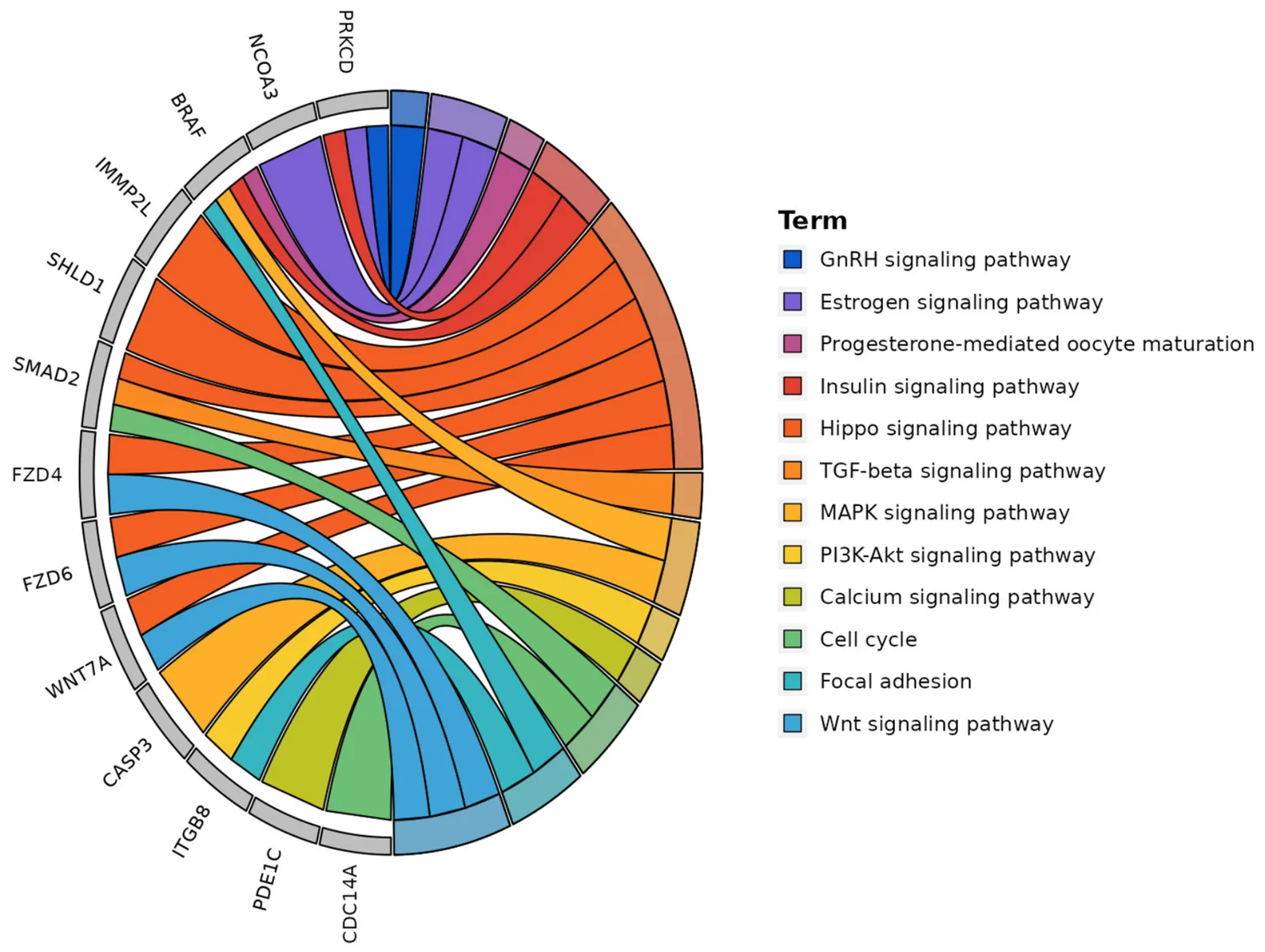
Gene-pathway enrichment chord diagram.

Functional enrichment analysis indicated that the target genes in this network were mainly enriched in signaling pathways such as the Estrogen signaling pathway, Progesterone-mediated oocyte maturation, and Hippo signaling pathway, all of which are known key pathways promoting follicle activation and estrus initiation (Figure 9).

**Figure 10:**
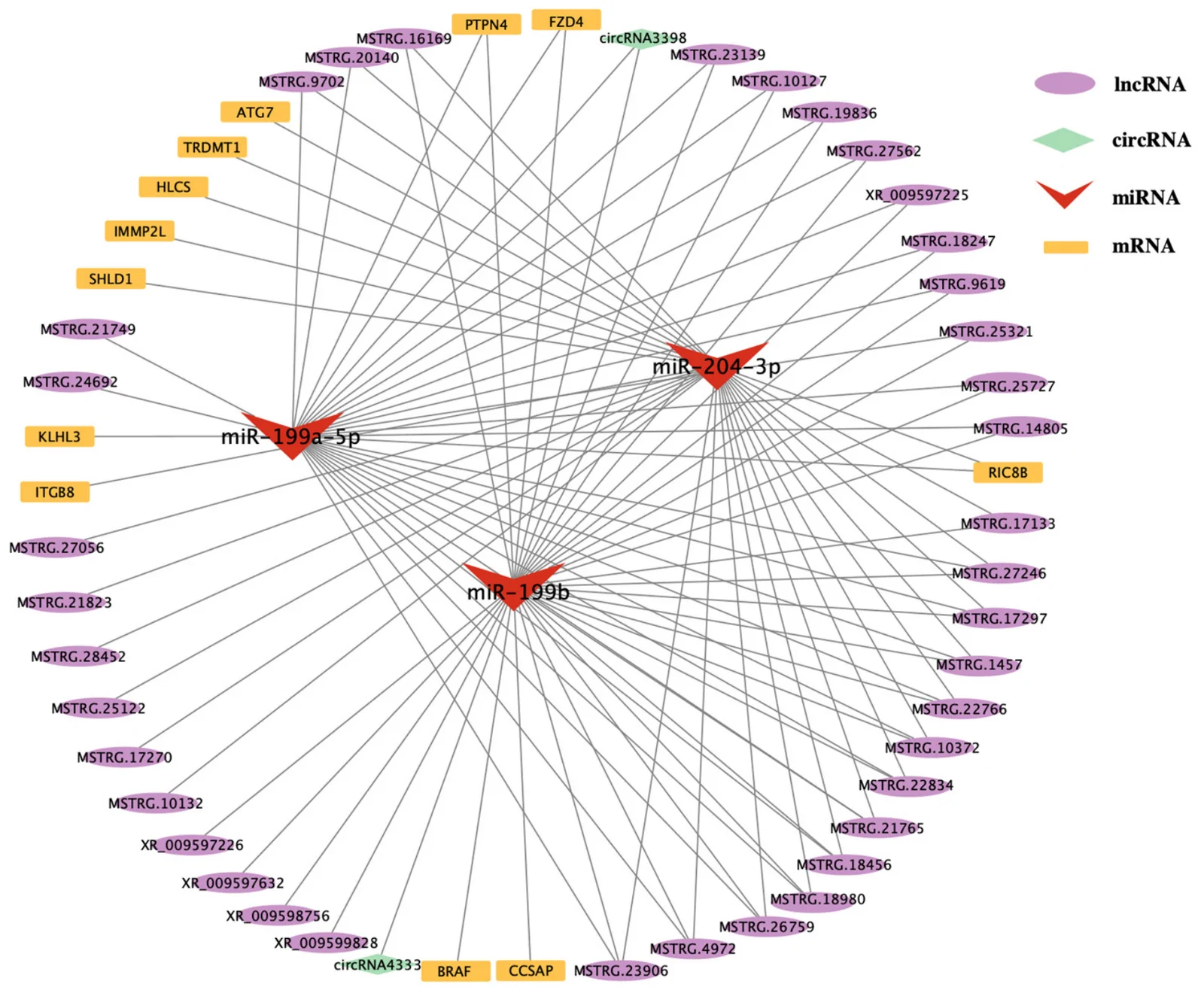
ceRNA regulatory network of core hub miRNAs.

Core regulatory axis analysis revealed that miR-186, oar-miR-136, and the miR-199 family occupied central positions in the network, with connectivity degrees significantly higher than those of other nodes, suggesting that these miRNAs and their upstream lncRNA/circRNA regulatory axes may constitute the “accelerator-type” driving core for estrus initiation in Small Tail Han sheep. Combined with the “brake-releaser” miRNA network, these two classes of regulatory factors synergistically form a dual “de-inhibition–activation” regulatory mode in response to the estrous cycle, jointly explaining the molecular basis of the permanent estrus trait in Small-tailed Han sheep. Network topology analysis identified miR-199a-5p, miR-204-3p, and miR-199b as high-connectivity candidate hub miRNAs in the predicted ceRNA network, as shown in Figure 10. However, this hub designation is derived solely from connectivity degree and does not establish causal regulation. Experimental validation is required to determine whether these miRNAs regulate their predicted targets and contribute to estrus regulation.

### 3.8. RT-qPCR Validation

**Figure 11:**
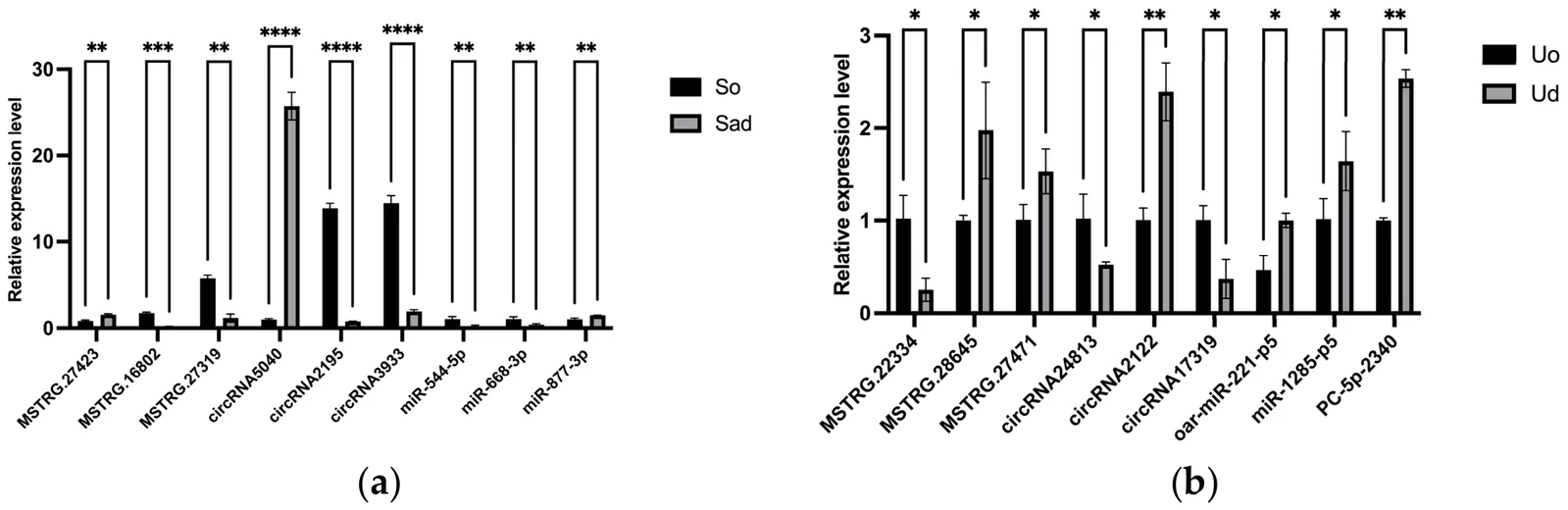
Validation of RT-qPCR: (**a**) So vs. Sad; (**b**) Uo vs. Ud (* *p* < 0.05, ** *p* < 0.01, *** *p* < 0.001, **** *p* < 0.0001).

To verify the reliability of the RNA-seq quantitative results, we randomly selected 3 DELs (So vs. Sad: MSTRG.27423, MSTRG.16802, MSTRG.27319; Uo vs. Uad: MSTRG.28645, MSTRG.22334, MSTRG.27471), 3 DECs (So vs. Sad: circRNA5040, circRNA2195, circRNA3933; Uo vs. Uad: circRNA24813, circRNA2122, circRNA17319), and 3 DEMs (So vs. Sad: miR-544-5p, miR-668-3p, miR-877-3p; Uo vs. Uad: oar-miR-221-p5, miR-1285-p5, PC-5p-2340) from the upregulated and downregulated differentially expressed molecules in each of the two comparison groups, totaling 18 candidate molecules for RT-qPCR analysis. The results showed that the expression trends of all 18 selected candidates in both comparison groups were consistent with the RNA-seq data (as shown in Figure 11), indicating that the differentially expressed profiles obtained in this study are highly reproducible and accurate.

## 4. Discussion

Sheep reproductive activity exhibits distinct breed-dependent differentiation: most sheep breeds are seasonally estrous, with their reproductive activity driven by photoperiodic changes and concentrated in specific seasons (e.g., autumn); however, the Small-tailed Han sheep has broken this seasonal restriction, displaying year-round (permanent) estrus regardless of season [28]. This phenotypic difference not only endows Small-tailed Han sheep with higher reproductive efficiency under intensive farming conditions but also makes it an ideal model for dissecting the regulatory mechanisms of the mammalian estrous cycle. Although previous studies have explored the genetic basis of sheep fecundity at the level of candidate genes (e.g., BMPR1B, GDF9, etc.) [29,30], a systematic understanding of the molecular regulatory logic underlying “permanent estrus” as a continuous trait—particularly regarding how non-coding RNAs function during the transition between estrus and diestrus—is still lacking. Moreover, compared with seasonally estrous breeds, the breed-specific regulatory strategies at the ovarian transcriptomic level in permanently estrous breeds remain to be elucidated, which limits our understanding of the molecular mechanisms behind the divergence of reproductive strategies in sheep.

In this study, we employed whole-transcriptome sequencing to systematically compare ovarian tissues of Small Tail Han sheep (permanently estrous) and Ujumqin sheep (seasonally estrous) during both estrus and diestrus, aiming to reveal the commonalities and differences in estrous response patterns between the two breeds at the level of ncRNA regulatory networks. We found that the number of differentially expressed transcripts involved in the estrus-to-diestrus transition in Small Tail Han sheep (3475) was substantially higher than that in Ujumqin sheep (906), with a particularly striking disparity at the mRNA level (1881 vs. 23). This magnitude of difference (nearly 82-fold) far exceeds the typical transcriptomic variation observed between breeds, suggesting that permanently and seasonally estrous breeds may fundamentally differ in how their ovaries decode estrous cycle signals. The near-”silence” at the mRNA level in Ujumqin sheep contrasts sharply with the widespread transcriptional reprogramming commonly observed in mammalian estrous cycle studies [31,32,33], implying that the ovaries of seasonally estrous breeds actively maintain a “transcriptomic rigidity” during cycle transitions. In contrast, the highly “transcriptomic plasticity” exhibited by Small Tail Han sheep ovaries may constitute the molecular basis for their ability to sustain cyclic ovulation outside the breeding season. This dichotomy offers a novel perspective on the permanent estrus trait—the critical question may not be “what permanently estrous breeds have gained” but rather “what seasonally estrous breeds have actively constrained.” On the other hand, we observed a cross-breed conserved pattern in the directional changes in different RNA types across the estrous cycle, which merits further discussion. circRNAs were predominantly upregulated in both breeds—485 (84.6%) of DECs in Small Tail Han sheep and 299 (80.2%) in Ujumqin sheep. Conversely, mRNAs and lncRNAs were mainly downregulated, with downregulated mRNAs accounting for 63.6% and downregulated lncRNAs for 64.2% in Small Tail Han sheep, and 65.2% for mRNAs in Ujumqin sheep. This paired pattern of “universal circRNA upregulation and universal mRNA downregulation” is unlikely coincidental. The massive accumulation of circRNAs during estrus may serve as miRNA sponges [32,34], competitively binding to and neutralizing inhibitory miRNAs, thereby indirectly “releasing the brake”—which precisely provides an upstream mechanistic explanation for the brake-releaser miRNA model subsequently proposed in this study. Meanwhile, the widespread downregulation of mRNAs suggests an alternative possibility: during estrus, ovaries may actively suppress most basal transcriptional activities, reallocating cellular resources to critical events essential for ovulation, such as cumulus expansion, oocyte meiotic resumption, and pre-ovulatory follicle wall remodeling. This transcriptional strategy of “streamlining the scope to concentrate on key breakthroughs” is manifested in both sheep breeds, indicating that it represents a conserved logic in the regulation of the estrous cycle.

Only four miRNAs—let-7a-3p, let-7a-5p, miR-320a, and miR-500—were identified as conserved core miRNAs shared between the two breeds. The paucity of this number is itself revealing: the miRNAs mobilized during the estrus-to-diestrus transition differ far more between breeds than they overlap. The target genes of these four conserved miRNAs were significantly enriched in the cAMP, PI3K-Akt, MAPK, and oocyte meiosis signaling pathways. The cAMP pathway acts as a central hub for gonadotropin signal transduction [35], PI3K-Akt directly modulates granulosa cell survival and follicle maintenance [36], and MAPK is essential for oocyte maturation [37]. These observations imply that, regardless of whether a breed is permanently or seasonally estrous, the ovary must accomplish a minimal set of core tasks during cycle transitions: rendering follicles responsive to gonadotropins and enabling oocytes to complete meiosis. Strikingly, although Ujumqin sheep displayed only 23 DEGs at the transcriptomic level, the differential expression of these four conserved miRNAs remained evident. This suggests that miRNA-level fluctuations alone may suffice to drive ovulation by exerting a disproportionately large effect on a limited set of mRNA changes—a “punching above their weight” phenomenon. Indeed, previous work has shown that even subtle changes in ovarian miRNAs can amplify downstream outputs through targeted regulation of a few key transcription factors [38], a notion that aligns well with our current findings.

Further comparison of estrous-responsive molecules between the two breeds identified two classes of breed-specific miRNAs—the brake-releaser and accelerator-type—whose biological significance lies in distinguishing two distinct regulatory strategies. The brake-releaser miRNAs (*n* = 38) were highly expressed during diestrus and markedly downregulated during estrus. During diestrus, when the ovary is functionally quiescent, these highly expressed miRNAs may maintain a “brake” state by suppressively targeting genes involved in follicle activation. Upon entry into estrus, these miRNAs are largely cleared, releasing their target genes from repression. This represents a “passive” mode of activation—lifting inhibition is itself activation. The accelerator-type miRNAs (*n* = 21), in contrast, were significantly upregulated during estrus and may indirectly amplify hormonal signals by targeting and suppressing negative regulators (e.g., inhibitors of the TGF-β pathway), reflecting an “active” driving mode. The coexistence of both strategies in the Small Tail Han sheep ovary carries important physiological implications. A pure “brake-release” strategy might yield incomplete or fluctuating estrus initiation, whereas “active activation” alone could be constrained by the background of inhibitory miRNAs accumulated during diestrus. The synergy between the two ensures the decisiveness and reliability of estrus onset. This dual strategy may well constitute the molecular safeguard that enables Small Tail Han sheep to sustain cyclic ovulation even outside the breeding season—when photoperiodic conditions are unfavorable for estrus initiation. By eliminating basal inhibition via brake-releaser miRNAs and reinforcing positive drive via accelerator-type miRNAs, the ovary becomes capable of mounting a sufficient response even to weak external signals.

The ceRNA network analysis further revealed a clear functional stratification between the pathways targeted by conserved core miRNAs and those targeted by accelerator-type miRNAs. The conserved core miRNA-associated ceRNA network converged on pathways such as cAMP, PI3K-Akt, and MAPK signaling—pathways that can be viewed as universal sensors for transducing external signals, translating fluctuations in gonadotropin levels into intracellular responses [39,40,41]. In contrast, the accelerator-type miRNA ceRNA network was specifically enriched in pathways directly involved in follicle maturation and ovulation execution, including estrogen signaling, progesterone-mediated oocyte maturation, and Hippo signaling. If the conserved network is responsible for “sensing,” then the accelerator network is tasked with “executing.” The permanent estrus phenotype of Small Tail Han sheep may therefore arise not from a heightened sensitivity to photoperiodic cues—given that the sensing module appears conserved across breeds—but rather from a greater capacity of the ovary to execute the ensuing responses. This executive capacity is conferred by breed-specific miRNAs and their upstream ceRNA regulatory networks. Of particular interest are miR-199 family members and miR-186, which occupy central positions in the accelerator network and may act as signal amplifiers bridging upstream hormonal signals with downstream follicular events. Notably, the Hippo signaling pathway has garnered increasing attention in ovarian biology, with evidence supporting its involvement in follicle activation and maintenance of the primordial follicle pool [42,43,44]; however, direct links between Hippo signaling and the regulation of the ovine estrous cycle remain scarce. The enrichment observed in our study points to a previously underappreciated role for Hippo pathway in estrus initiation, thereby incorporating this pathway into the potential core regulatory circuitry of the sheep estrous cycle and providing a new candidate pathway for dissecting the functional differences between seasonally and permanently estrous breeds at the ovarian execution level. Our findings also resonate with earlier transcriptomic studies on the sheep estrous cycle. The importance of FoxO, Wnt, and cAMP signaling pathways in ovarian follicular development and cycle regulation has been repeatedly corroborated in multiple previous investigations [45,46,47]. The consistent enrichment of these pathways in both breeds in our dataset not only attests to the reliability of our sequencing data but also furnishes a solid foundation for the subsequent discussion.

In summary, this study proposes a “ncRNA-mediated transcriptomic plasticity” hypothesis for the permanent estrus trait in sheep: the molecular basis of permanent estrus in Small Tail Han sheep does not reside in a single key gene mutation or overexpression, but rather in the ovary’s maintenance of a higher level of dynamic transcriptomic responsiveness. This capacity is conferred by a multi-layered ceRNA network composed of circRNAs and lncRNAs, and operates through the synergistic actions of brake-releaser and accelerator-type miRNAs. Through integrated analysis of both ceRNA networks, we ultimately pinpointed miR-199a-5p, miR-204-3p, and miR-199b as core hubs bridging the two regulatory modules, which may serve as candidate molecular markers for genomic selection of reproductive traits in sheep. This work provides a novel molecular framework for understanding how the ovary decodes and amplifies intrinsic signals through non-coding RNA networks to drive cyclic ovulation, and also furnishes an actionable set of candidate molecules for genetic improvement of the permanent estrus trait in sheep.

This study has several limitations. The sample size was limited to three biological replicates per group, and no a priori power analysis was performed. Subtle changes may therefore have been missed, and the results should be regarded as hypothesis-generating. Future studies should validate the candidate miRNAs and ceRNA axes in larger cohorts, with a priori power analysis and, where possible, multi-batch or multi-center sampling. FecB genotyping was not performed, and individual lambing records were unavailable. Because Small Tail Han sheep are both year-round estrous and highly prolific, and because FecB (BMPR1B) status and parity can influence ovarian transcriptomes, fecundity- and parity-related effects cannot be fully separated from estrus cyclicity. Future work should genotype FecB, record parity and lambing performance, and compare ovarian transcriptomes within each genotype across estrus and diestrus. Cycle stage was determined by behavioral estrus detection after CIDR + PMSG synchronization, without serum hormone measurements; future studies should combine behavioral observation with progesterone, estradiol, LH, and FSH measurements to confirm staging more rigorously. Long and small RNA libraries were sequenced on different platforms, but each platform included all four groups, so platform effects were not confounded with breed or estrous stage; future unified-platform sequencing would help confirm the key findings, and absolute expression levels across RNA types should not be directly compared. Finally, the ceRNA networks and hub miRNAs are computational predictions. Future studies should prioritize functional validation of the highest-connectivity axes, such as circRNA13828–let-7a-5p–PRKCD and miR-199a-5p with its estrogen-pathway targets, using dual-luciferase reporter assays, miRNA overexpression/knockdown, and in vitro follicle or granulosa cell models. These limitations define the scope of our conclusions: the identified miRNAs and ceRNA axes are candidate regulators rather than established mechanisms.

## 5. Conclusions

In this study, we compared the ovarian transcriptomes of year-round estrous Small Tail Han sheep and seasonal estrous Ujumqin sheep during estrus and diestrus. The two breeds exhibited markedly different transcriptional response strategies. The number of differentially expressed mRNAs in Small Tail Han sheep was 82-fold that in Ujumqin sheep, indicating greater “transcriptomic plasticity” in the year-round estrous breed. Based on expression dynamics, we identified 38 “brake-releaser” miRNAs and 21 “accelerator” miRNAs, which together form a dual “de-repression–activation” regulatory mode. Among them, miR-199a-5p, miR-204-3p, and miR-199b were identified as core hubs connecting the two regulatory modules.

From the perspective of genetic improvement, this study provides candidate molecules for marker-assisted selection or genomic selection. The core miRNAs (miR-199a-5p, miR-204-3p, and miR-199b) and the conserved core miRNAs (let-7a-3p, let-7a-5p, miR-320a, and miR-500) can serve as candidate markers for the year-round estrus trait. More importantly, the “de-repression–activation” dual model suggests that breeding programs can select in two directions: first, selecting for low-expression alleles of brake-releaser miRNAs to reduce diestrus-associated inhibition of follicular activation; second, selecting for high-expression alleles of accelerator miRNAs to enhance the positive drive of estrus initiation. Core ceRNA axes, such as circRNA13828–let-7a-5p–PRKCD, can be prioritized for marker development.

For researchers, this study provides several testable regulatory hypotheses. The Hippo signaling pathway, estrogen signaling pathway, and progesterone-mediated oocyte maturation pathway are priority targets for functional validation, especially the role of Hippo signaling in ovine estrus initiation, which has received little attention. Core ceRNA axes, such as circRNA13828–let-7a-5p–PRKCD and miR-199a-5p together with its estrogen-pathway target genes, should be validated first using dual-luciferase reporter assays and RNA interference/overexpression experiments. Such work will convert the current expression-correlation-based predictions into functional evidence.

The main limitation of this study is that the ceRNA networks are computational predictions and have not yet been experimentally validated. Future work should focus on the highest-connectivity core axes, validate their regulatory functions in in vivo and in vitro models, and further evaluate the breeding value of these candidate markers in different sheep populations. Overall, this study provides concrete candidate molecules and an actionable research path for breaking the seasonal breeding constraint in sheep and enabling year-round balanced production.

## Data Availability

The raw RNA-seq data generated in this study have been deposited in the NCBI Sequence Read Archive (SRA) under the BioProject accession number PRJNA1289050 and PRJNA1253307.

## Supplementary Materials

The following supporting information can be downloaded at: https://www.mdpi.com/article/10.3390/ani16182925/s1, Figure S1: Flowchart of fr-firstrand library construction; Figure S2: Flowchart of RNase R+ library construction; Figure S3: Schematic diagram of BSJ recognition; Figure S4: Classification statistics chart of lncRNAs; Figure S5: Expression profile characteristics of lncRNAs; Figure S6: Expression profile characteristics of circRNAs; Figure S7: Expression profile characteristics of miRNAs; Figure S8: Volcano plot of differentially expressed ncRNAs and mRNAs in So vs. Sad; Figure S9: Volcano plot of differentially expressed ncRNAs and mRNAs in Uo vs. Ud; Table S1: RT-qPCR primers sequence information; Table S2: Detection results of total RNA quality; Table S3: mRNA, lncRNA and circRNA sequencing data production quality; Table S4: miRNA sequencing data production quality.

## Abbreviations

The following abbreviations are used in this manuscript:

ncRNA	non-coding RNA
DEMs	differentially expressed miRNAs
DELs	differentially expressed lncRNAs
DECs	differentially expressed circRNAs
DEGs	differentially expressed mRNAs
HPOA	hypothalamic-pituitary-ovarian axis
GnRH	gonadotropin-releasing hormone
FSH	follicle-stimulating hormone
LH	luteinizing hormone
