# Supplementary material for: Whole-Transcriptome Landscape Deciphers a ceRNA-Mediated “De-Repression–Activation” Circuit Governing Continuous Estrus in Ovine
Source: Animals (Basel). 2026 Sep 17;16(18):2925. doi: 10.3390/ani16182925 (PMC13603349; doi:10.3390/ani16182925)
Supplement: Supplementary file 1 [file animals-16-02925-s001.zip › animals-4554020-supplementary.pdf]

Table S1 RT-qPCR primers sequence information

Genes	Primer sequences(5'→3')	Fragment length/bp
β-actin	F: GATCTGGCACCACACCTTCTA	115
	R: GATCTGGGTCATCTTCTCACG	
MSTRG.27423	F:AAACTGCGTGGGTTGCGAGAG	149
	R: AACTCTGGTGGAGGTCCGTAGC	
MSTRG.16802	F: GCGTTGCTCTGTTCTTTATCTCCTG	144
	R: ACCTGTGCTGTCTGAGTCTTGTG	
MSTRG.27319	F: CTGCCTTCTTGTCCACTGCTTTG	148
	R:GGTGACGCTGCCATCATTGATATG	
circRNA5040	F:CAAAACGTGAAGAAATAATGGAGA	206
	R:CACATTTGGATCCAACAACTGATG	
circRNA2195	F:GCTTTGAATGGTGTGGAGGATG	208
	R:CGCAGAGGGTCTGGGAGAAG	
circRNA3933	F:GGAAGACACTCTGTGCTTTATGGC	213
	R:GTTGGTTAAGGGTATTTGTGCATC	
miR-544-5p	F:GTCGTATCCAGTGCAGGGT	19
miR-668-3p	F:GTCGTATCCAGTGCAGGGT	19
miR-877-3p	F:GTCGTATCCAGTGCAGGGT	19
U6	F:TGAAGCGTGCTCGCTTCGGC	89
MSTRG.28645	F: GGTCGCTCGCTCCTCTCCTAC	128
	R: GAGGCTGACCGGGTTGGTTTTG	
MSTRG.22334	F:CCTGCATGGAGCTGGTGTTGG	117
	R:CGCCCAGGTCACCCACTAGG	
MSTRG.27471	F:CCTGATCTTGTCCATCGTCCTATCC	123
	R:GGGAACTGACCAAGGATGATAAGGG	
circRNA24813	F:CACAGAGTCAAGAGACTAACGGGC	179
	R:CTCTCCAACTTCCACAATTTGCTC	
circRNA2122	F: TTGATCCAGAATTCTCCTGAGTGC	190
	R:GTTGACTATATAATTGTTGAGAAAT	
circRNA17319	F:TGAAGAACGAGCAGTAATTCTAGG	273
	R:AATGAAAATAAAGATATTTTATTGC	
oar-miR-221-p5	F:GTCGTATCCAGTGCAGGGT	19
miR-1285-p5	F:CGGATCAGTAGTGGGATCGC	19
PC-5p-2340	F:ACGAGATCCTCCTGACTCCAGG	19

Table S2 Detection results of total RNA quality

Samples	concentration (μg/μL)	RIN	OD value(260/280)
So_1	0.17	7.40	1.87
So_2	0.06	7.80	1.98
So_3	0.09	9.70	1.95
Sad_1	0.38	8.50	1.96
Sad_2	0.31	8.60	1.86
Sad_3	0.24	7.20	1.89
Uo_1	0.05	7.20	1.90
Uo_2	0.04	7.30	1.83
Uo_3	0.04	7.90	1.92
Ud_1	0.49	8.20	1.83
Ud_2	0.05	7.50	1.89
Ud_3	0.07	10.00	2.00

Table S3 mRNA、lncRNA and circRNA sequencing data production quality

Sample	Raw Data	Valid Data	Mapping rate（%）	Q20/%	Q30/%	GC content%
So_1	74,106,726	71,910,234	66,210,782(92.07%)	99.85	98.05	45.00
So_2	90,402,358	87,502,414	81,890,808(93.59%)	99.82	97.99	44.50
So_3	88,799,682	86,496,168	81,411,487(94.12%)	99.86	98.02	45.00
Sad_1	94,092,386	91,107,390	86,158,306(94.57%)	99.96	97.78	45.00
Sad_2	97,790,686	94,803,474	89,533,252(94.44%)	99.96	97.71	45.00
Sad_3	89,825,602	86,701,034	80,875,819(93.28%)	99.97	98.16	48.50
Uo_1	92,231,148	89,013,622	83,650,569(93.98%)	99.81	98.31	44.50
Uo_2	95,944,996	92,841,144	86,837,283(93.53%)	99.81	98.24	46.00
Uo_3	89,602,454	86,517,238	81,071,259(93.71%)	99.79	98.16	46.00
Ud_1	73,320,580	70,898,902	65,359,324(92.19%)	99.85	97.83	47.00
Ud_2	92,249,184	87,955,300	82,065,791(93.30%)	99.77	97.86	50.50
Ud_3	71,516,612	68,959,698	63,802,517(92.52%)	99.81	97.60	48.00

Table S4 miRNA sequencing data production quality

Sample	Raw Data	Valid Data	Mapping rate（%）	Q20/%	Q30/%	GC content%
So_1	11,994,726	7,224,597	7,153,073(99.01%)	99.26	97.11	51.19
So_2	11,533,015	6,679,309	6,629,214(99.25%)	99.29	97.16	51.05
So_3	12,845,516	5,824,807	5,763,647(98.95%)	99.16	97.11	51.78
Sad_1	9,796,916	6,971,109	6,898,609(98.96%)	97.92	96.91	51.40
Sad_2	10,567,624	7,951,578	7,863,315(98.89%)	97.92	96.96	51.22
Sad_3	10,036,758	6,868,427	6,794,248(98.92%)	97.29	96.07	51.11
Uo_1	10,902,739	6,614,309	6,564,040(99.24%)	99.26	97.16	51.08
Uo_2	11,917,122	6,735,685	6,679,105(99.16%)	99.36	97.49	51.20
Uo_3	11,925,958	7,321,518	7,265,874(99.24%)	99.33	97.42	51.10
Ud_1	11,082,057	6,789,313	6,743,146(99.32%)	99.24	97.13	51.14

Ud_2	11,408,596	7,210,142	7,160,392(99.31%)	99.26	97.10	50.87
Ud_3	11,172,807	7,387,622	7,321,133(99.10%)	99.28	97.18	50.94

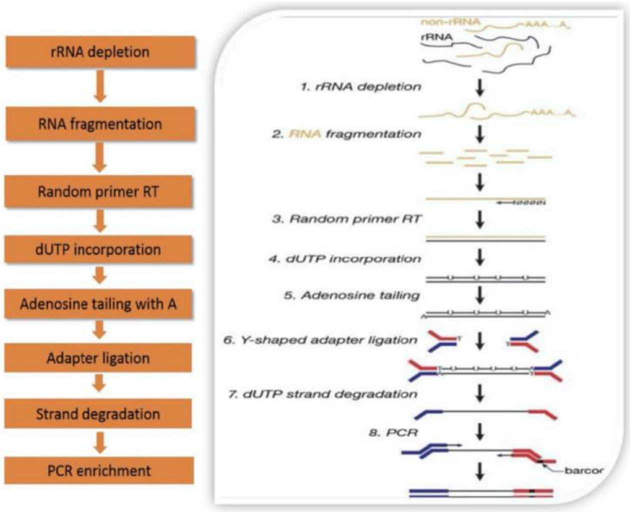

Figure S1. Flowchart of fr-firstrand library construction

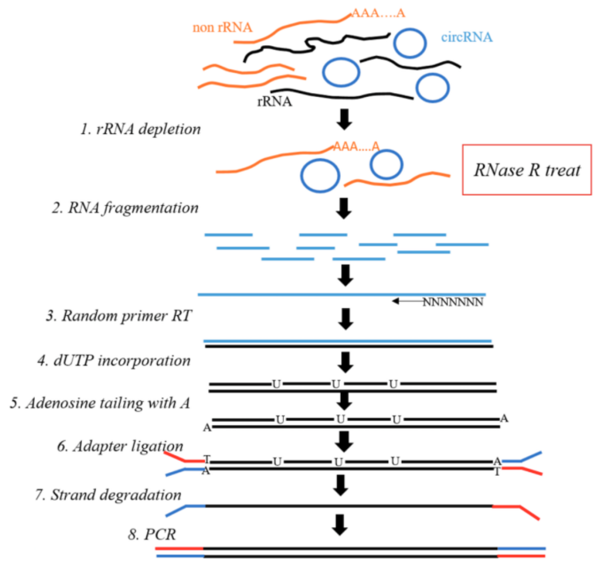

Figure S2. Flowchart of RNase R+ library construction

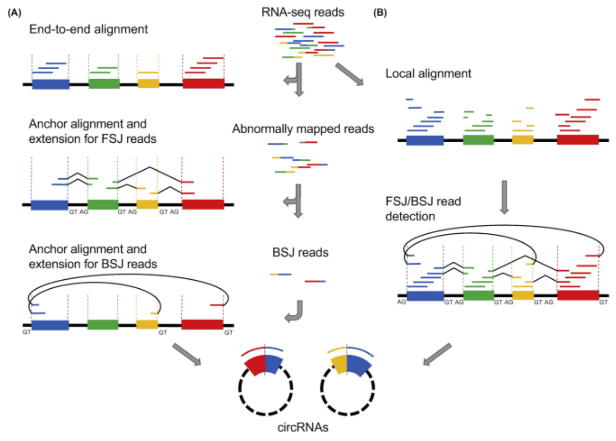

Figure S3. Schematic diagram of BSJ recognition

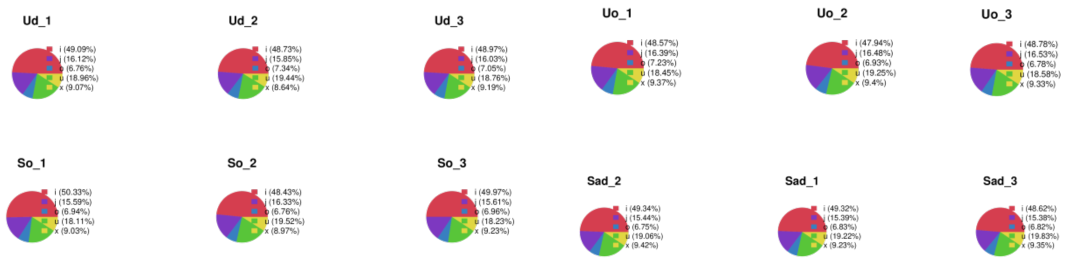

Figure S4. Classification statistics chart of lncRNAs

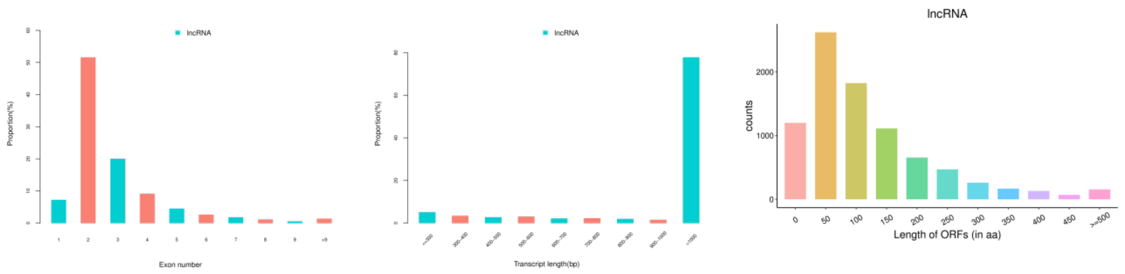

a b c

Figure S5. Expression profile characteristics of lncRNAs

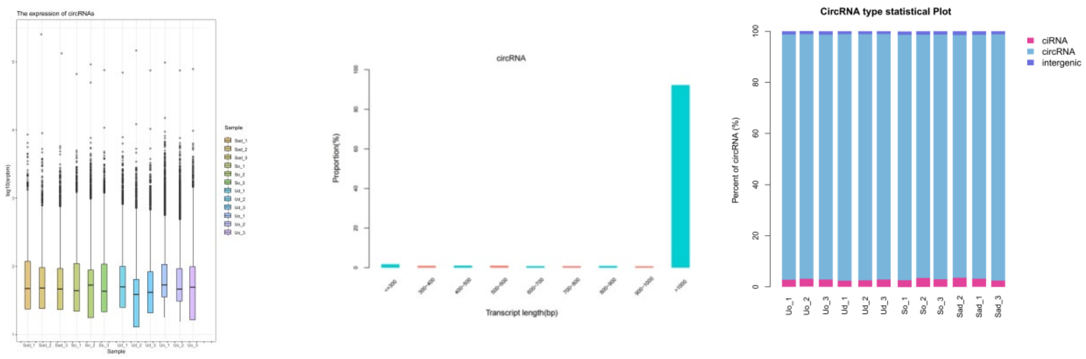

a b C

Figure S6. Expression profile characteristics of circRNAs

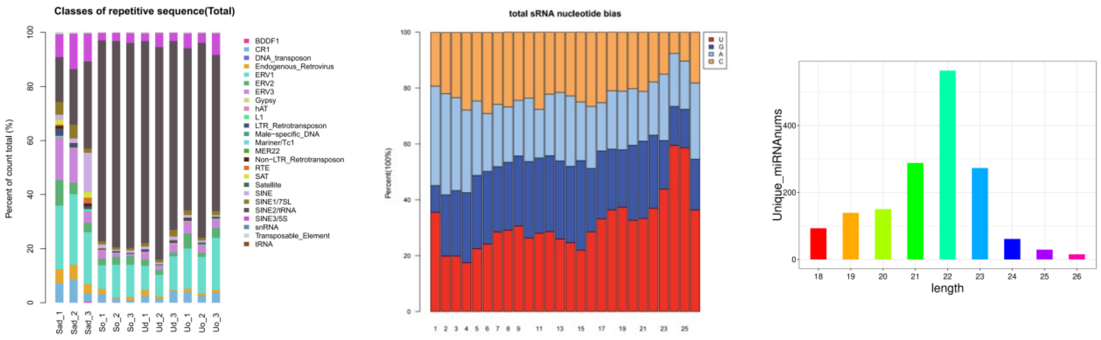

a b c

Figure S7. Expression profile characteristics of miRNAs

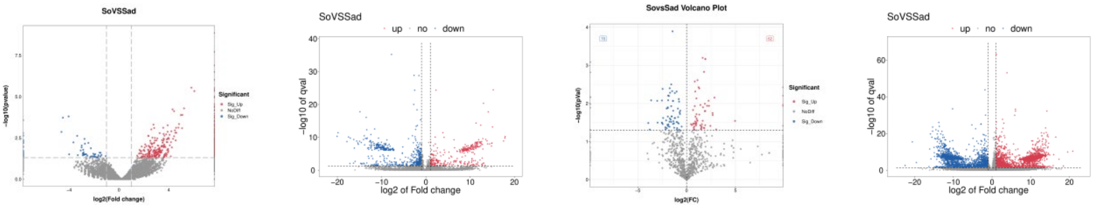

a b c d

Figure S8. Volcano plot of differentially expressed ncRNAs and mRNAs in So vs. Sad

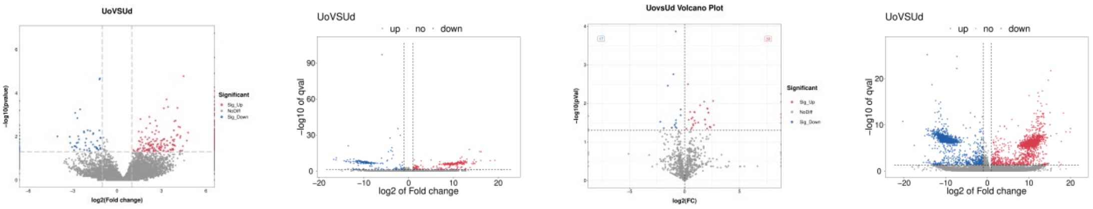

a b c d

Figure S9. Volcano plot of differentially expressed ncRNAs and mRNAs in Uo vs. Ud
